# Evaluating the diagnostic pathway for acute ACL injuries in trauma centres: a systematic review

**DOI:** 10.1186/s12891-022-05595-0

**Published:** 2022-07-07

**Authors:** Natasha E. H. Allott, Matthew S. Banger, Alison H. McGregor

**Affiliations:** Sir Michael Uren Hub, Imperial College London, White City Campus, 86 Wood Lane, London, W12 0BZ UK

**Keywords:** ACL, Diagnosis, Acute injury, Emergency department, Care pathway

## Abstract

**Objective:**

This review sought to evaluate the literature on the initial assessment and diagnostic pathway for patients with a suspected Anterior Cruciate Ligament (ACL) tear.

**Methods:**

MEDLINE, EMBASE, and CINAHL were systematically searched for eligible studies, PRISMA guidelines were followed. Studies were included if they used at least one assessment method to assess for ACL injury and participants were assessed at an acute trauma centre within 6-weeks of injury. Article quality was evaluated using the QUADAS-2 checklist.

**Results:**

A total of 353 studies were assessed for eligibility, 347 were excluded for the following reasons: injuries were not assessed in an acute trauma setting, injuries were not acute, participants had previous ACL injuries or chronic joint deformities affecting the knee, participants were under 18, or participants included animals or cadavers. A total of six studies were included in the review. Common assessment methods included: laxity tests, joint effusion, inability to continue activity, and a history of a ‘pop’ and ‘giving way’ at the time of injury. Diagnostic accuracy varied greatly between the assessment method and the assessing clinician. Gold standard diagnostics were MRI and arthroscopy. A weighted meta-mean calculated the time to reach diagnosis to be 68.60 days [CI 23.94, 113.24]. The mean number of appointments to reach diagnosis varied from 2–5. Delay to surgery or surgical consultation ranged from 61 to 328 days.

**Conclusion:**

Clinicians in the Emergency Department are not proficient in performing the assessment methods that are used for diagnosis in acute ACL injury. Reliance on specialist assessments or radiological methods inevitably increases the time to reach a diagnosis, which has repercussions on management options. There is an ever-growing demand to improve diagnostic accuracy and efficiency; further exploration into quantitative measures of instability would aid the assessment of peripheral joint assessment.

## Introduction

Musculoskeletal complaints make up 30% of primary care consultations in the UK [1], with acute knee injuries accounting for approximately 5–8% of all acute injuries seen in the Accident and Emergency unit (A&E) [2, 3]. Since the anterior cruciate ligament (ACL) is the main contributor to preventing tibia-femoral anterior translation and provides stability during rotation, the clinical assessment of acute ACL injury consists of laxity tests which assess for anterior and rotary stability of the knee, such as: Lachman’s, anterior draw, and the pivot shift test. Clinical history and mechanism of injury can also act as a primary diagnostic indicator [4, 5]. ACL injuries are debilitating, for this reason, early diagnosis is key to facilitating efficient treatment outcomes.

Acute ACL injury is renowned to be difficult to assess and it is frequently missed by clinicians on initial assessment [6, 7]. Most acute ACL injuries present in trauma settings, such as A&E departments and minor injury units [3, 8, 9]. During the acute injury phase, joint effusion and muscular compensation are barriers to assessment [3, 8] where excessive joint effusion is postulated to reduce diagnostic accuracy to 12.7% [3]. Clinical assessment methods, whilst low cost, are subject to significant errors; diagnostic accuracy can be as low as 56% [6]. A paper published in 1996 stated diagnosis takes on average 21 months [10], a more recent paper exploring the assessment of ACL’s in the emergency department concluded there has been little improvement since then, with only 14.4% of ACL injuries diagnosed at initial presentation [6].

Emergency physicians have poor diagnostic accuracy, as low as 25.9%, compared to more experienced professionals such as sports medicine physicians when assessing acute ACL injury [8, 11]. However, Perera’s study emphasised that even with specialist training, orthopaedic physicians missed diagnosis in 28% of patients [8]. This reinforces that clinicians cannot ensure diagnostic accuracy with the current clinical assessment methods, thus emphasising the importance of more accurate diagnostic tests readily available at acute trauma centres to improve patient outcomes [11].

Early diagnosis is paramount as it can reduce the likelihood of the knee giving way, which is associated with secondary injury, specifically to the meniscus which can result in osteochondral damage [12]. Unfortunately, up to 50% of ACL injuries lead to post-traumatic osteoarthritis (PTOA) [13, 14]. ACL injuries lead to an increase in anterolateral rotary instability [15], such instability, unless corrected by surgical or conservative means, leads to degradation on bony and cartilaginous structures which contributes to the progression of arthritic changes within the joint [16]. The British Orthopaedic Association not only highlights the importance of accurate, fast diagnosis, but it also recommends that any ACL injury is referred to a surgeon as soon as possible to facilitate optimal patient outcomes, be that surgical or conservative management [16].

Understanding current assessment methods and diagnostic pathways for patients with acute ACL tears is paramount to developing more efficient diagnosis recommendations and assessment measures. This article aims to systematically review the literature for assessing patients with suspected ACL tears that come in through the emergency department.

## Materials and Methods

The PRISMA checklist was followed throughout in order to ensure a robust review process was followed.

### Search strategy

The databases MEDLINE, EMBASE, and CINAHL were used to conduct the search, published articles up until the 25th of May 2021 were eligible for inclusion in the review. The search terms were either classed as headings or keywords. The Boolean search strategy was used to appropriately combine terms and retrieve the most relevant articles (Table 1). ‘ACL’ AND ‘trauma centre’ were the two topic groups, all keywords and subject headings within the topic heading ‘ACL’ were combined with ‘OR’, this was repeated with the keywords and subject heading within the topic groups ‘trauma centre’. Topic groups were then combined with ‘AND’. After the automated search using the terms listed in Table 1, a hand search was undertaken which cross-referenced the terms in Table 1 against the bibliographies of the relevant articles.

**Table 1:** Boolean search strategy

Topic Group	Subject headings		Keywords
ACL	MEDLINE EMBASE	Anterior cruciate ligament/	ACL OR anterior cruciate Adj2 ligament OR anterior adj2 cruciate ligament
		Exp Anterior cruciate ligament rupture/	
		Exp Anterior cruciate ligament injury/	
	MEDLINE OVID	Anterior cruciate ligament/	
	EBSCO CINAHL	MH “anterior cruciate ligament”	
Trauma center	MEDLINE EMBASE	Emergency ward/	Emergency department* OR emergency health service* OR emergency adj2 accident OR first contact practitioner* OR casualty OR triage* OR delay adj2 diagnosis OR late* adj1 diagnos* OR orthop?edic clinic* OR knee clinic* OR Emergency ward*
		Exp emergency health service/	
		Delayed diagnosis/	
	MEDLINE OVID	EXP Emergency medical services/	
		Delayed diagnosis/	
	EBSCO CINAHL	MH “emergency medical services+”	
		MH “diagnosis, delayed”	

### Eligibility criteria

The review scoped the current assessment methods and diagnostic pathways for ACL injury in the emergency department (ED) for patients. For articles to be eligible for the systematic review, they had to meet the following inclusion criteria: the assessment had to be completed in an Emergency Department, injury was acute on initial assessment (within 6 weeks), at least one clinical assessment tool was stated, and participants were deemed to have sustained an ACL injury. Articles were excluded if: participants had experienced a previous ACL injury, participants had known chronic joint deformities (such as osteoarthritis), animals or cadavers were included within the sample, the paper was not available in English, and if participants were under the age of 18. Study designs that were case reviews, case studies or series were also excluded.

### Selection process

All articles identified from the search from the databases were exported to a reference manager software called Endnote X20 (Clarivate Analytics, Philadelphia, PA, USA). Duplicates were removed, all references were then exported to another reference management software called Covidence (Veritas Health Innovation, Melbourne, Australia). Articles were first screened by text and abstract, those who were deemed relevant underwent a full-text review. The articles were screened against the inclusion-exclusion criteria by two independent reviewers (N.A and A.M), discrepancies between reviewers were then resolved by a discussion.

Studies that included participants under the age of 18 were contacted asking for data specific to the population of interest. A few articles did not specify whether the injury was acute or chronic, thus the authors of these articles were contacted for further information.

### Data extraction

The following data were extracted from the selected articles: Aim; sample size; study design, time since injury, participant number, clinical assessment tool, reference standard, assessing clinician (profession and experience), time to reach diagnosis, the median number of appointments to reach diagnosis, the meantime to await surgery and diagnostic accuracy.

### Statistical analysis

A meta mean with associated confidence intervals was calculated for the outcome: time to reach diagnosis.

### Quality appraisal

The QUADAS-2 checklist (Appendix 1) was the quality appraisal assessment tool was used to assess papers. The QUADAS-2 is widely used for studies evaluating diagnostic accuracy assessment measures. It is made up of 4 domains: patient selection, index tests, reference standards, and flow and timing. As shown in Table 2, the first 3 domains are split into assessing sections A and B, A) risk of bias, and B) concerns regarding applicability. The 4th domain: flow and timing, did not include section B. The sections were comprised of signalling questions (Table 2) that would ultimately determine the ‘risk of bias’ and ‘concern regarding applicability’. The QUADAS-2 is not designed to give an overall quality score, instead, it provides an overall judgment on the two assessment criteria. If a paper had more than one signalling question that was ‘at high risk’ (X) it was regarded as ‘at risk of bias’, the ‘concern regarding applicability’ was then assessed.

**Table 2:** Signalling questions for QUADAS-2 quality assessment

Signaling questions for QUADAS-2 quality assessment	
**Domain 1: patient selection**	
A: risk or bias	Was a consecutive or random sample of patients enrolled?
	Did the study have appropriate exclusions?
	Was the study retrospective?
	Was a sufficient sample size used?
	**Could the selection or patient have introduced bias?**
B: concerns regarding applicability	**Are there concerns that the included patients do not match the review question?**
**Domain 2: index test(s)**	
A: risk or bias	Were the index test results interpreted without knowledge of the results of the reference standard?
	Were all the index tests specified and clearly explained?
	**Could the conduct or interpretation of the index test have introduced bias?**
B: concerns regarding applicability	**Is there concern that the index test, its conduct, or interpretation differ from the review question?**
**Domain 3: reference standard**	
A: risk or bias	Is the reference standard the ‘gold standard’ for ACL diagnosis?
	Were the reference standard results interpreted without knowledge of the results of the index test?
	**Could the reference standard, its conduct, or its interpretation, have introduced bias?**
B: concerns regarding applicability	**Is there concern that the target condition as defined by the reference standard does not match the review question?**
**Domain 4: flow and timing**	
A: risk of bias	Was there an appropriate interval between the index test and the reference standard?
	Was the time frame defined where the initial consultation (index test) and/or reference standard was completed? /Unclear
	Did all patients receive a reference standard?
	Did all patients receive the same reference standard?
	Were all patients included in the analysis?
	**Could the patient flow have introduced bias?**

## Results

**Fig. 1:**
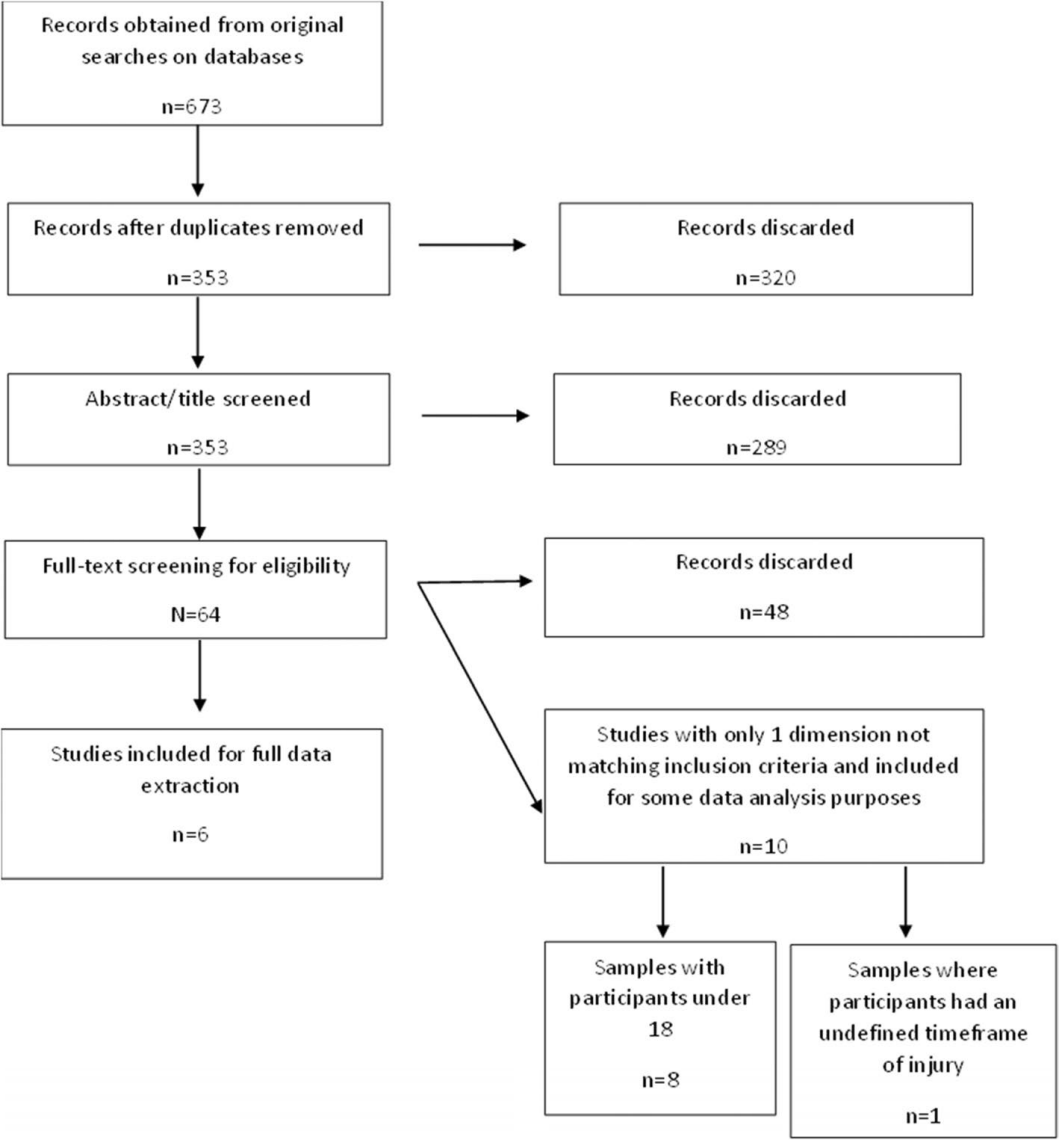
PRISMA diagram detailing the selection process

A total of 673 articles were initially obtained across all three databases. After duplicates were removed, 353 articles were included in the review. Once the titles and abstracts were screened, 64 articles were eligible for a full-text review. Following this, six articles were identified as being appropriate for inclusion, Fig. 1 details the PRISMA diagram and the reasons for article exclusion at each stage.

### Main characteristics of included studies

Table 3 details the main characteristics of the included studies of the review. The study completed by Clifford et al. [9] included participants with injuries that were outside of the acute timeframe (6 weeks), authors provided raw data for all participants and only the data of those who matched the inclusion criteria (61/82) were included in this review.

**Table 3:** Main characteristics of included studies

Author (Date)	Article title	Journal	Aims	Participants	Protocol
**Ball et al (2010)** [12]	The impact of an Acute Knee Clinic	Annals of the Royal College of Surgeons of England	Evaluate the impact of an acute knee clinic on diagnosis and treatment for acute knee injures	100	**Design:** • Prospective study • Consecutive sampling **Methods:** • Audit or timeframes and assessment process
**Clifford et al (2021)** [9]	Acute knee clinics are effective in reducing delay to diagnosis following anterior cruciate ligament injury	Knee	Investigate the impact of an acute knee clinic compared to the standard A&E pathway	61 (that matched the inclusion criteria)	**Design:** • Prospective study • Consecutive sampling **Methods:** • Physical examination • Imaging • Arthroscopy
**Hardy et al (2017)** [3]	The use of history to identify anterior cruciate ligament injuries in the acute trauma setting: the ‘LIMP index’	Emergency Medicine Journal 2017	To investigate what clinical history features indicate ACL injuries	194 (163 available)	**Design:** • Prospective study • Consecutive sampling **Methods**: • Survey • Questionnaire • Physical examination • Imaging
**Lee and Yun (2019)** [17]	Feasibility of point-of-care knee ultrasonography for diagnosing anterior cruciate and posterior cruciate ligament tears in the ED	American Journal of Emergency Medicine 2019	To evaluate the use of ultrasound compared to MRI to diagnose ACL injury	62	**Design:** • Prospective study • Consecutive sampling **Methods:** • Physical examination • Imaging
**Parwaiz et al (2016)** [6]	Anterior cruciate ligament injury: A persistently difficult diagnosis	Knee	To investigate if there has been an improvement in ACL diagnosis over the last 20 years	160	**Design:** • Retrospective design • Consecutive sampling **Methods:** • Retrospective data extraction
**Wang et al (2016)** [7]	Efficacy of knee joint aspiration in patients with acute ACL injury in the emergency department	Injury 2016	To evaluate the impact of joint aspiration on the sensitivity of joint laxity tests on patients with ACL injuries through the emergency department	60	**Design:** • Retrospective design • Consecutive sampling **Methods:** • Retrospective data extraction • Physical examination • Imaging

### Article quality

The QUADAS-2 showed variation between studies, as represented in Table 4. The risk of bias in domain 1 was high for 50% of the articles [6, 7, 12], these were negatively impacted due to an insufficient sample size attributable to the absence of power calculations, inappropriate exclusion criteria, and a retrospective design including only patients who underwent arthroscopy.

**Table 4:** Methodological quality summary

STUDY	RISK OF BIAS				APPLICABILITY OF CONCERNS		
	Domain 1: PATIENT SELECTION	Domain 2: INDEX TEST	Domain 3: REFERENCE STANDARD	Domain 4: FLOW AND TIMING	Domain 1: PATIENT SELECTION	Domain 2: INDEX TEXT	Domain 3: REFERENCE STANDARD
Ball et al. 2010 [12]	X	X	?	?	✓	?	?
Hardy et al. 2017 [3]	✓	✓	X	X	✓	✓	✓
Lee and Yun 2019 [17]	✓	✓	✓	✓	✓	✓	✓
Wang et al. 2016 [7]	X	✓	✓	?	✓	✓	✓
Parwaiz et al. 2016 [6]	X	X	✓	?	✓	?	✓
Clifford et al. 2021 [9]	✓	✓	✓	X	✓	✓	✓

KEY:

✓ = LOW

X = HIGH

? = UNCLEAR

**Fig. 2:**
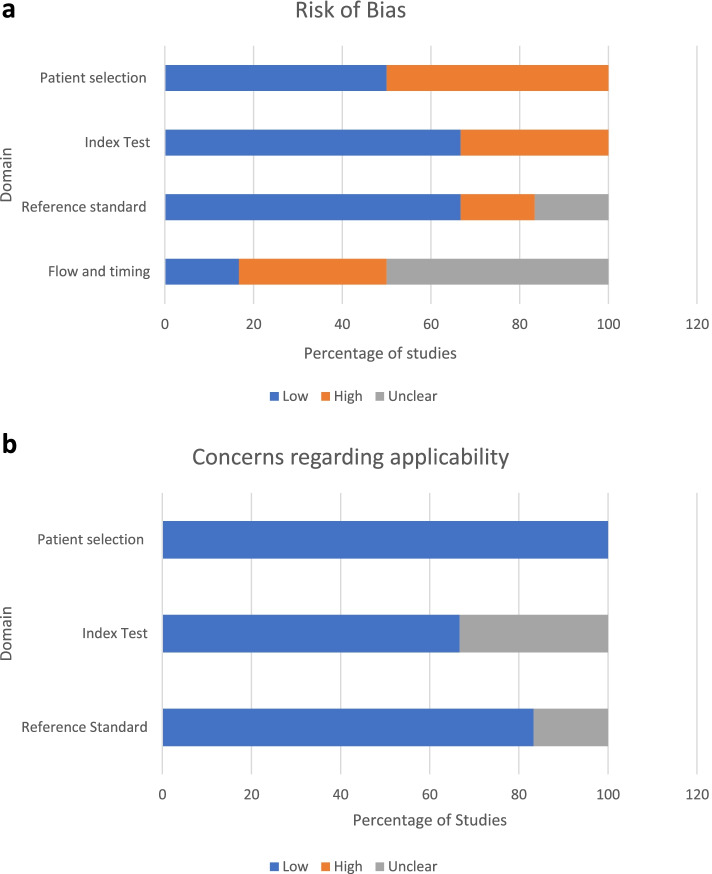
**a** Risk of bias summary. **b** Concerns regarding applicability summary. **a** and **b**: methodological quality graph summary: Bar chart representing the percentage of studies that are rated low, high, and clear for each domain for both sections **A** (risk of bias) and **B** (concerns regarding applicability)

Other factors that seemed to affect studies quality assessment were: insufficient description of their assessment methods [6, 12], injury verification using means that are not considered ‘gold standard’ for diagnosis [3], undefined timeframes between initial assessment and reference standard [6, 7, 12], and finally the inconsistent use of an injury verification method [9, 12]. Figure 2 represents the percentage of articles scoring high, low, and unclear for each domain.

### Index tests

Table 5 presents the index tests and reference standards used in the studies included in the review. An index test, in this instance, is the initial assessment method(s) used to evaluate the knee. Passive laxity tests such as Lachman’s, anterior drawer, and the pivot shift test were used to assess knee instability in two of the articles [7, 9]. Lee and Yun [17] used ultrasound and an unspecified clinical assessment as their index test. Joint effusion was part of the initial assessment in 3 of the studies [3, 7, 9], inability to continue activity was also an injury indicator in two of these articles [3, 9]. Another study’s index tests comprised of palpation, temperature, and joint line tenderness [2], whilst a different article used X-Rays as a means of ruling out fractures [7]. Giving way and a ‘pop’ at the time of injury were a strong indicator of injury in two of the articles [9]. The other two studies left their initial assessment method unspecified [7, 9].

**Table 5:** Assessment methods

AIM	TEST	INDEX TEST	REFERENCE STANDARD
Laxity	Lachman’s	(7, 9)	[9]
	INSTABILITY’	–	–
	Lever sign	–	–
	Pivot shift	(7, 9)	[9]
	Anterior drawer	(7, 9)	[9]
	KT1000	–	–
Range of movement	Active range of movement	(7)	–
	Passive range of movement	–	–
Swelling	Time delay swelling	(9)	–
	Joint effusion	(7, 9)	[9]
Functional ability	Weight bare	–	–
	Inability to continue activity	(9)	–
	Gait	–	–
Pain	Palpation	(7)	–
	Temperature	(7)	–
	Joint line tenderness	(7)	–
	Pain	–	–
Imaging	X-RAY	(9)	[9]
	Ultrasound	(17)	–
	CT	–	–
	MRI	–	[3, 9, 17]
Subjective assessment	Mechanism of Injury	–	–
	Lysholms functional score	–	–
	Locking	–	–
	Unspecified clinical history	(3, 6)	–
	Clicking	–	–
	Giving way	(9)	–
	Popping sound	(9)	–
	Limp index	(3, 9)	–
Unspecified assessment	Unspecified clinical assessment	(6, 17)	[3]
	Physicians’ agreement	–	–
	Unspecified	(12)	[6, 12]
	Orthopaedic surgeon exam	–	–
Surgery	Anaesthetic eval	–	–
	Arthroscopy	–	[3, 7, 9]

### Reference standard

A reference standard, in the context of ACL injury, is a verification tool used to measure the accuracy of the respective index test. MRIs were used as a reference standard in many of the studies [3, 9, 17, 18]. Whereas, other participants were retrospectively recruited from ACL reconstructive surgeries, leaving arthroscopy as the designated reference standard [3, 7, 9]. In Clifford et al’s study [9], only four participants did not have an MRI, of the 61 patients that met the inclusion criteria, 25 were initially diagnosed using MRI alone, another 25 were diagnosed from clinical examination findings, 9 participants diagnoses were confirmed from a combination of MRI and clinical assessments, and 2 had an arthroscopy. Hardy et al. [3] verified their index test with either an unspecified clinical assessment, MRI, or arthroscopy. They did not specify which participants had the respective reference standard [3]. Some studies did not specify their diagnosis verification, they simply eluded that ACL injury was confirmed [6, 12].

### Time to reach diagnosis

**Fig. 3:**
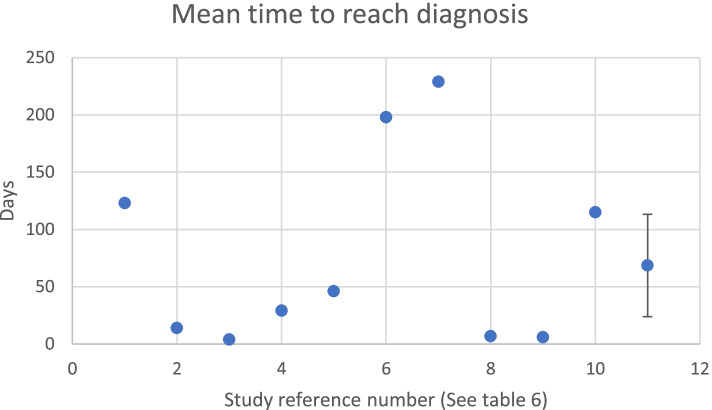
Scatter Diagram showing the ‘meantime to reach diagnoses for individual sub-sample groups (Table 6), and meta mean with 95% confidence intervals

Figure 3: meantime to reach diagnoses for individual sub-sample groups.

Table 6 represents the time taken to reach diagnosis, this varied between studies. Whilst Ball et al. [12] found that the implementation of an acute knee clinic reduced the time to reach diagnosis from 123 to 14 days. Lee and Yun’s participants [17] were immediately scheduled for an MRI after their ultrasound on initial presentation to A&E, they reported diagnosis took an average of 3.8 days. Hardy et al. [3] observed a considerable increase in the delay to diagnosis when follow-ups were not scheduled, ranging from 29 to 198 days, when the patients who were diagnosed at initial consultation were removed this increased further to 229 days. Wang et al’s [7] participants were all referred to an out-patient department to be seen by professionals in a specialist knee clinic within 7–14 days of initial presentation to the ED. It was eluded that confirmation of diagnosis was reached at this outpatient visit, patients who had an aspiration performed on initial presentation took 6.7 days to be diagnosed; those who did not, took an average of 6 days. Parwaiz et al. [6] had 48.8% of the participants present to A&E, of these, only 5% were diagnosed on initial presentation, there was no further data on how long diagnosis took to be confirmed. Clifford et al’s data revealed diagnosis took on average 115 days [9].

**Table 6:** Time to reach diagnosis

Author	Study components	Study reference number	Meantime to reach diagnosis (days)	Sample size	Mean number of appointments to reach diagnosis
Ball et al. 2010 [12]	Before acute knee clinic (AKC)	1	123	100	5
	Post-AKC	2	14	100	1
Lee and Yun 2019 [17]	Ultrasound	3	3.8	62	2
Hardy et al. 2017 [3]	Follow up arranged	4	29	120	–
	Follow up arranged with initial diagnosis removed	5	46	101	
	No follow up arranged	6	198	43	
	No follow up arranged with initial diagnosis removed	7	229	40	
Wang et al. 2016 [7]	Aspirated knee group’	8	6.7	18	–
	Non-aspirated	9	6.0	42	
Parwaiz et al. 2016 [6]	Those presenting to an Emergency Department	–	Not documented – although only 5% reached initially	78	–
Clifford et al. 2021 [9]	Whole Participant sample	10	115	61	3.3
**Meta Mean**		**11**	**68.60**	–	–

Table 6 showing the allocated study reference number for the sub-study components, including sample size, the meantime to reach diagnosis, and the mean number of appointments to reach diagnosis

#### Statistical analysis

A weighted mean time to reach diagnosis yielded a result of 68.60 days [CI 23.94, 113.24] (Fig. 3).

### Mean number of appointments to reach diagnosis

Three of the papers reported the number of appointments to reach diagnosis, ranging from 2 to 5 (Table 6). However, the number of appointments varied depending on the study design, those that only used imaging modalities took less time to reach diagnosis (2 appointments) [17] compared to those that did not (3.3 and 5 appointments) [12, 9].

### Assessing clinician

Table 7 displays the assessing clinicians and time until surgery. Ball et al. [12] did not specify the assessing clinician before the acute knee clinic was established. Patients assessed in the acute knee clinic were seen by a ‘knee specialist’. The study by Lee and Yun [17] included a board-certified emergency physician with over 5 years of experience, and an MSK radiologist familiar with ultrasonography and its affiliation with ACL injury. The radiologist trained the emergency department practitioner in sonography prior to the study [17]. Hardy et al. [3] described the assessor in the acute knee clinic as ‘a person highly trained in a particular branch of medicine’, 22 were included, some of which were surgeons. Wang et al’s [7] initial clinical assessment was performed by a junior orthopaedic surgeon; they were then followed up by a senior orthopaedic surgeon with over 10 years of experience [7]. Parwaiz and Clifford et al’s patients were assessed by a variety of clinicians on initial presentation [6, 9].

**Table 7:** Tabulation of assessing clinician and delay to surgery

Author	Assessing clinician		Time until surgery	
**Ball et al. 2010** [12]	Unspecified		196	
	‘specialist’		126	
**Lee and Yun. 2019** [17]	Board-certified emergency physician with over 5 years of experience AND specialist MSK radiologist		–	
**Hardy et al. 2017 (time to initial appointment with surgeon)** [3]	22 specialists		Follow up arranged	61
			Follow up arranged with initial diagnosis removed	69
			No follow up arranged	328
			No follow up arranged with initial diagnosis removed	311
**Wang et al. 2016** [7]	In the ED	junior orthopaedic surgeon	–	
	In the OPD follow-up	senior orthopaedic surgeon		
**Parwaiz et al. 2016** [6]	advanced nurse practitioner OR A junior ED trainee OR A senior ED trainee		–	
**Clifford et al. 2021** [9]	triaged by an A&E Sister and assessed by an A&E physician	1	–	
	A&E registrar	1		
	triaged by nurses and assessed by an A&E Physician	4		
	normal adult triage route and then assessed by an A&E physician	53		
	adult triage and was then assessed by an emergency nurse practitioner	1		
	orthopaedic doctor	1		

### Time until surgery

Not all studies included surgery data (Table 7). Those that did ranged from 61–328 days [3, 12]. Factors that increased time were not having an established acute knee clinic [12], not having a follow up arranged after initial consultation [3], and removing the cohort who were diagnosed on initial consultation [3].

### Diagnostic accuracy

Values of diagnostic accuracy were not available for all studies due to the way participants were recruited; Table 8 demonstrates the values of diagnostic performance characteristics. Lee and Yun [17] recorded high accuracy of both ultrasound performers 1 and 2 of 91.9 and 93.6% respectively. Three of the studies only had values of true positive, and false negative cases; the participants that were assessed were of a population of confirmed ACL tears, meaning only sensitivity data was available [3, 7, 9]. In order for specificity, accuracy, PPV and NPV to be calculated, false positive and true negative data must be available [19, 20]. Hardy et al. [3] recorded that 99.5% of participants reported at least 1 facet of the LIMP index, 95.8% reported 2, 83.9% of participants reported 3, this dropped to 57.8% of participants reporting all 4 variables of the LIMP index. The Lachman’s test yielded poor accuracy in Wang et al’s study when initially performed in the Emergency Department at the time of injury in both the aspirated and non-aspirated cohort (47.1 and 40.5% respectively). This increased to 76.5% in the aspirated cohort when reviewed 2 weeks later in the outpatient department, this value was similar to the sensitivity reported by Clifford et al. (74.5%). Values for the pivot shift test varied massively (9.5–100%), the accuracy increased significantly in the aspirated knee group (from 11.8 to 76.5%). Clifford et al’s study revealed an unremarkable sensitivity of 100%, however, this result was only based on 3 participants. Parwaiz et al. [6] did not have any data on the diagnostic accuracy of their assessment methods.

**Table 8:** Diagnostic accuracy of index tests

Authors	Diagnostic accuracy						
**Ball et al. 2010** [12]	Unspecified						
**Lee and Yun 2019** [17]			**Ultrasound performer 1 (%)**		**Ultrasound performer 2 (%)**		
	**Sensitivity**		90.6		96.9		
	**Specificity**		93.3		90		
	**Positive predictive value (PPV)**		93.6		91.2		
	**Negative Predictive value (NPV)**		90.3		96.4		
	**Accuracy**		91.9		93.6		
**Hardy et al. 2017** [3]	**Sensitivity**	**All 4 facets**	57.8%				
		**3 facets**	83.9%				
		**2 facets**	95.8%				
		**1 facet**	99.5%				
**Wang et al. 2016** [7]		**Group 1- Aspirated knee (Sensitivity)**			**Group 2- non-Aspirated knee (Sensitivity)**		
		ED	Lachman’s	47.1%	ED	Lachman’s	40.5%
			Pivot shift	11.8%		Pivot shift	9.5%
		OPD	Lachman’s	76.5%	OPD	Lachman’s	47.5%
			Pivot shift	76.5%		Pivot shift	31.0%
**Parwaiz et al. 2016** [6]		Unspecified					
**Clifford et al. 2021** [9]				**Sensitivity**			
		**Lachman’s**		74.5%^a^			
		**Anterior Drawer**		61.4%^a^			
		**Pivot Shift**		100%^a^			

Table 8 Table showing the accuracy, sensitivity, specific NPV and PPV of assessment methods. The performance characteristics were dependent on the data available in the articles

^a^Values were re-calculated from the raw data sent by the authors. Only the population of interest were included in these values. Participants outside of the 6-week timeframe, under 18, or assessed outside of A&E were excluded

## Discussion

This literature review evaluated the current diagnostic methods and assessment pathways available to patients presenting themselves to an Emergency Department with a suspected ACL injury. Although numerous papers investigated diagnostic accuracy for ACL tears, only 6 met the inclusion criteria for this review.

A reoccurring theme in the literature, is that the time to reach a diagnosis is reliant on a thorough assessment at initial presentation, which is highly dependent on the proficiencies of the clinician in question. The difficulty arises when inexperienced assessors are presented with an acutely swollen knee [6, 7, 21], indicating a greater need for a quantifiable measure of instability amongst non-specialist departments, in order to reduce prolonged and false-negative diagnoses.

False positives and false negatives are the two obstacles within acute MSK injury assessment, if assessment criteria are too rigid and specific, false negatives occur; patients are discharged without the ACL injury identified [9, 22], which is a direct cause of lengthy diagnoses. Contrary to this, if assessment criteria are too comprehensive, although accuracy may be high, false-positive diagnoses will also be in abundance, thus encompassing many other differential diagnoses and overwhelming the service; a prime example of this is within Hardy et al’s LIMP index [3].

Of the 6 papers reviewed, many articles had high risk of bias, this was primarily due to: (a) absence of power calculations, (b) retrospective design (c), unspecified index tests and reference standards, and (d) undefined timeframes between diagnostic tests and verification. Many studies were insufficiently powered or were retrospective in nature, sampling patients who had undergone reconstruction is not representative of the whole population. Both factors increase the risk of bias for domain 1. Unspecified index tests and reference standards lead to an inability to assess the risk of bias or concern regarding applicability, thus affecting the quality scores of domains 2 and 3. Domain 4 was negatively impacted by undefined timeframes between the initial assessment (index test), and the diagnostic verification (reference standard). If this is not standardised amongst participants, longer wait times between the index test and reference standard will have repercussions on the recorded accuracy of the assessment method. Consequently, this quality review has identified many areas for improvement for studies evaluating diagnostic tests.

A plethora of index tests were used to identify ACL tears. Laxity tests were common and often accompanied by a subjective assessment and a history of the mechanism of injury. Swelling alluded to a high suspicion of an ACL tear, however it was also a barrier to accurate assessment, as demonstrated by Wang et al. [7]. The authors theorised that aspiration reduces Substance P within the joint, which consequently decreases pain and prevents the patient from ‘guarding’ during joint instability tests [7].

When performed by a specialist clinician, the diagnostic performance characteristics of clinical tests increase significantly [7] this is reflected by the inconsistencies amongst pivot shift test sensitivity (9.5–100%) [7, 9]. Diagnostic rates during initial assessment within the emergency department are reported as low as 5% [6], this is attributable to few clinicians performing stability tests during their assessment, often secondary to an unconfident skill set. Instead, patients of concern are triaged to specialist practitioners. Although this triaging pathway ensures a more accurate assessment, relying solely on specialists to undertake initial assessments relies on Emergency Department clinicians to be able to recognise those suspicious of ACL injury appropriately.

Radiological methods, such as ultrasound and X-rays [9, 17] were popular index tests. Ultrasound allows for fast comparison to the contralateral limb and is not subject to lengthy waitlists [17]. Although found useful in this review, ultrasound is not routinely used in the Emergency Department to assess for ACL injury due to the lack of available specialists in front door services [17].

X-rays were used as an assessment adjunct to rule out bony pathology. Nearly all studies used either MRI or arthroscopy to verify injury [11, 23–28] and these were the most popular assessment verification method [3, 7, 9, 17]. As such, both are considered as gold standard diagnostic approaches [29, 30], many clinicians within these studies would only confirm a diagnosis on their completion [9, 17], suggesting that many healthcare professionals are not confident in confirming diagnosis based on clinical assessment alone.

Although the findings of this paper reflect that the diagnostic accuracy of clinical assessment has high degrees of variability (Table 7), diagnostic accuracy of clinical assessments has been reported consistently high in other papers [31–35]. Though it must considered that the majority of findings in such papers are based on injuries that are no longer in the acute time frame [31, 33, 34], which minimises the obstacles that are associated with acute assessment [7, 9]. In addition, the assessors were often highly experienced in assessing acute knee injury [33], and the respective diagnostic accuracy values were reported in a primary [32], or secondary care [31] setting, or they were performed under anaesthesia [35]; this is not reflective of front door assessing practitioners that complete the initial assessment screening process in the ED.

The varied time periods to reach a diagnosis is attributable to the differences in the study design and the assessing clinician. The weighted mean (68.60 days) for the time to reach diagnosis is not necessarily representative of clinical practice. Diagnostic performance characteristics were highest in the studies using ultrasound and joint aspiration [7, 17], both of which are unsurprising due to the expertise required to undertake these assessment methods.

There is a clear correlation between faster diagnoses and more efficient diagnostic pathways. Late diagnosis leads to delayed surgical review [3, 12], as illustrated in Table 7; this impacts the optimal management pathways. Those with clinical follow-ups arranged reached diagnosis in a shorter period [3, 7, 12]. Lengthy diagnoses were a consequence of prolonged referral times between initial assessment and a specialist review, resulting in multiple appointments before a diagnosis is reached [12] (Table 6). This is secondary to ineffective initial assessments and subsequent false negatives on clinical testing.

The effects of lengthy diagnosis are exposed over time, ACL deficient knees are associated with an increase in “giving way” episodes and knee instability. Whilst it has not yet been confirmed that an earlier diagnosis leads to a decreased chance in developing PTOA, it is not naïve to assume that earlier intervention would directly improve patient outcomes. ACL tears give rise to rotational instability and varus thrust [16, 36], those with a varus thrust have a 3-fold increase in developing osteoarthritis, furthermore those with defined OA are often ACL deficient or severely dysfunctional [16, 36]. Receiving the appropriate management, whether surgical or conservative, aims to prevent “giving way” and to correct gait abnormalities, which in turn, prevent subsequent injury deterioration and development of PTOA.

Limitations must be considered when analysing the review. Some of the included papers had high risk of bias, thus affecting the evidence of conclusions. Most of the data outcomes showed heterogeneity, subsequently a meta-analyses could not be completed. The time to reach diagnosis is hard to definitively timestamp, clinical impressions and working diagnoses are often made early on, the question therefore arises: when is diagnosis reached? Also, it must be considered that the ‘time taken until surgery’ was noted as an outcome for 2 of the studies, this is not representative of the conservative management group; complexities arise when trying to define a point in time to represent delayed diagnosis within this patient group. The knee is the most commonly injured joint within sport, with 90% of knee injuries involving the ACL [37]. This study only reflects a small proportion of these, more reviews like this must be undertaken for more data to be available and represented in this area. However, it must be recognised that the lack of publications in this area are a consequence of the adversities in setting up trails in acute settings and phases of injury. Finally, excluding participants under the age of 18 reduced our sample size, though this was appropriate for this review as this was only considering those who are skeletally mature and within an adult healthcare pathway, further reviews into the paediatric population would be of use to evaluate repercussions of late diagnoses in the younger population.

## Conclusion

Clinical tests are highly subjective in assessing acute knee injury. A&E clinicians are often not proficient in performing instability tests which can lead to inappropriate triaging of patients with an ACL injury. Radiological verification is unsurprisingly accurate; however, it is unattainable to seek immediate radiological verification for this patient group. ACL tears lead to inherent knee instability, if appropriate management Is not sought efficiently, this can lead to an increased chance of developing PTOA. Further research should begin to investigate methods of instability quantification in order to tackle the repercussions of delayed diagnoses.

## Data Availability

All data generated or analysed during this study are included in this published article.

## Abbreviations

ACL: Anterior cruciate ligament
A&E: Accident and emergency
ROM: Range of movement
TOI: Time of injury
PTOA: Post-traumatic osteoarthritis
MRI: Magnetic resonance imaging
MSK: Musculoskeletal
